# An Efficient Forest Smoke Detection Approach Using Convolutional Neural Networks and Attention Mechanisms

**DOI:** 10.3390/jimaging11020067

**Published:** 2025-02-19

**Authors:** Quy-Quyen Hoang, Quy-Lam Hoang, Hoon Oh

**Affiliations:** Department of Electrical, Electronic and Computer Engineering, University of Ulsan, Ulsan 44610, Republic of Korea; hqquyen@gmail.com (Q.-Q.H.); quylam925@gmail.com (Q.-L.H.)

**Keywords:** convolutional neural networks, object detection, forest fire detection, backbone network, attention mechanisms

## Abstract

This study explores a method of detecting smoke plumes effectively as the early sign of a forest fire. Convolutional neural networks (CNNs) have been widely used for forest fire detection; however, they have not been customized or optimized for smoke characteristics. This paper proposes a CNN-based forest smoke detection model featuring novel backbone architecture that can increase detection accuracy and reduce computational load. Since the proposed backbone detects the plume of smoke through different views using kernels of varying sizes, it can better detect smoke plumes of different sizes. By decomposing the traditional square kernel convolution into a depth-wise convolution of the coordinate kernel, it can not only better extract the features of the smoke plume spreading along the vertical dimension but also reduce the computational load. An attention mechanism was applied to allow the model to focus on important information while suppressing less relevant information. The experimental results show that our model outperforms other popular ones by achieving detection accuracy of up to 52.9 average precision (AP) and significantly reduces the number of parameters and giga floating-point operations (GFLOPs) compared to the popular models.

## 1. Introduction

Forest fires often cause enormous damage to human life and the environment [1]. The main reason for the great damage is that forest fires spread quickly before they are detected, making them difficult to extinguish. This paper considers the evolution of the existing vision-based model to effectively detect forest smoke, the early sign of a forest fire. The proposed model is designed based on convolutional neural networks (CNNs) and an attention mechanism, focusing on increasing accuracy and reducing the computational complexity of smoke plume detection.

According to the survey papers by Chowdary and Gupta [2], Alkhatib [3], and Barmpoutis et al. [4], many forest fire detection methods have been proposed. Early methods relied on fire lookout towers and tools like the Osborne Fire finder [5]; however, they were not effective due to continuous human intervention and potential human error. Some methods used sensors that can detect the signs of a fire outbreak, such as increased temperature, smoke, flames, or a lack of oxygen; they faced the challenge of reliably collecting data from sensors deployed across vast forested areas [6]. They also suffered from delayed fire detection because the fire alarm did not sound until the fire detection parameter values reached a preset threshold.

Recently, the direction of research has been shifting toward a vision-based approach that relies on artificial intelligence [7]. Existing vision-based approaches can be broadly divided into the following two categories: the image processing approach and the CNN-based approach. The former relies on image processing techniques to explore fire and smoke characteristics such as color, shape, and motion. Chen et al. [8], Vipin [9], and Yuan et al. [10] used RGB, YCbCr, and Lab color models, respectively, to extract fire and smoke pixels. Zhang et al. [11] used wavelet and fast Fourier transform methods to analyze the contours of the fire area in videos. Foggia et al. [12] combined the properties of color, shape, and motion using a multi-expert framework to increase detection accuracy. One recent approach utilized background subtraction and color segmentation to detect regions containing motion [13]. Since these approaches do not use high computational power, they may be suitable for devices with limited computational power, such as drones or surveillance cameras. However, to achieve a reasonable level of accuracy, they require careful image pre-processing steps and may need the use of different feature extraction algorithms for forest fire images in different situations.

In contrast, CNN-based approaches use deep learning techniques to automatically extract features from different images. Wang et al. [14] proposed a lightweight forest fire detection model by replacing the backbone network of YOLOv4 [15] with MobileNetv3 [16]. YOLOv4 is a popular object detection model known for its accuracy and speed, while MobileNetv3 is a lightweight convolutional neural network (CNN) designed to reduce computational load, making it suitable for resource-constrained devices. This method significantly reduces the computational load but comes with a trade-off in detection accuracy. Jiao et al. [17] used YOLOv3 [18] to detect forest fires with the utilization of an unmanned aerial vehicle (UAV) that could capture high-resolution videos and images. However, it did not work well for small smoke plumes or fires. Another approach developed by Zhang et al. [19] tried to detect forest smoke using Faster R-CNN [20]; although they improved the accuracy to some extent, there were some shortcomings in terms of the diversity of the forest fire images included in the dataset used in the experiment. Vani [21] employed Inceptionv3 [22] to train satellite images for forest fire detection. The problem with this satellite-based approach was that it could only capture large-scale fire images after the fire had spread over a large area. Furthermore, since Inceptionv3 only returned a fire or non-fire decision without boxing the fires, it required an extra step to determine the regions of the fires, which would take time and effort. One recent approach introduced by Meena et al. [23] used R-CNN [24] for forest fire detection. The high computational complexity of this model hindered its portability to monitoring devices. In summary, the existing approaches have made limited improvements in detection accuracy because they use popular models such as the YOLO series and Faster R-CNN as they are. Moreover, they often require a high computational load.

Based on the discussion so far, this paper introduces a forest smoke detection model featuring a new backbone architecture that is customized to increase the accuracy of smoke detection and reduce computational load. The proposed backbone is designed to effectively extract smoke features. By extracting object features through different views using kernels of varying sizes, it can better detect smoke plumes of different sizes. Furthermore, by using the depth-wise convolution of coordinate kernels, it can not only better extract the features of smoke plumes spreading along the vertical dimension but also reduce the computational load. Finally, by using an attention mechanism, it can focus on the important features of an image. As a result, the proposed model could achieve up to 52.9 average precision (AP), which far exceeds the accuracy of other models such as RetinaNet [25], YOLO [26,27,28], Faster-RCNN [20], and SSD [29], while significantly reducing the number of parameters and GFLOPs.

The rest of the paper is organized as follows: Section 2 presents the background; Section 3 describes the model architecture in detail; and Section 4 analyzes the experimental results and is followed by the conclusion in Section 5.

## 2. Background

### 2.1. Overview of Forest Fire Detection Model

**Figure 1:**
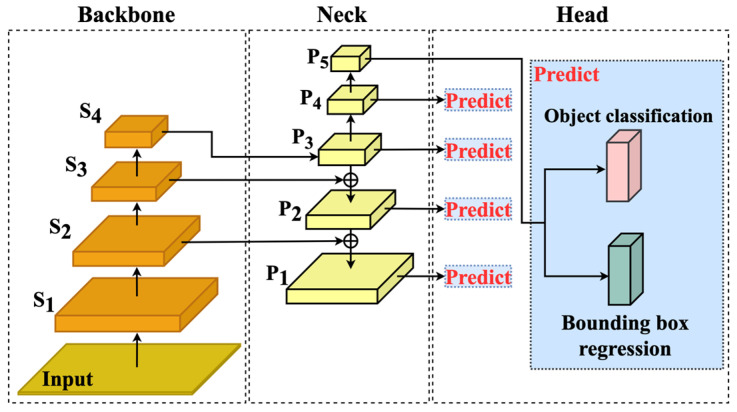
The architecture of the forest fire detection model.

The forest fire detection model consists of the following three modules: Backbone, Neck, and Head, as shown in Figure 1. The Backbone module has four stages labeled $S_{1},S_{2},S_{3}$, and $S_{4}$, each of which generates one feature map from the feature map of the stage below it, while $S_{1}$ generates a feature map from the input image. Early stages tend to capture low-level information such as edges, corners, etc., while later stages tend to capture higher-level or specific information.

The Neck and Head modules were defined by Lin et al. [25]. Neck has five levels labeled $P_{1},\;P_{2},\;P_{3},P_{4},$ and $P_{5}$, each of which has one feature map. The level feature map of $P_{3}$ is built by applying convolutions to the stage feature map of $S_{4}$; the level feature map of $P_{2}$ is created by up-sampling the level feature map of $P_{3}$ and adding it to the stage feature map of $S_{3}$; and the level feature map of $\;P_{1}$ is created similarly. Note that the level feature map of $P_{3}$ is used to generate the level feature map of $P_{4}$. Two more level feature maps of $P_{4}$ and $P_{5}$ are constructed by down-sampling those of $P_{3}$ and $P_{4}$, respectively, to have more abundant features. In this way, using a multi-level pyramidal network [30], Neck can not only balance the information via multiple stages but also help the model easily detect objects of different scales. Head consists of the following two primary components: object classification and bounding box regression. The object classification component predicts the class to which an object belongs, assigning a probability score to each class. The bounding box regression component, on the other hand, estimates the coordinates of the bounding box that encloses the detected object. These two components work together to increase the accuracy of object identification within an image.

### 2.2. Motivation and Our Approach

Backbone plays an important role in determining the accuracy of object detection, as it creates a feature map of the object. However, many convolutional layers may be involved, resulting in significant computational load.

Recent forest fire detection models have utilized a well-known backbone designed on the ImageNet dataset [31]. Unfortunately, ImageNet does not have smoke and fire classes. This means that those backbones were not optimized for forest fire and/or smoke detection. In addition, ImageNet is a large dataset with over one million images and one thousand classes. Thus, researchers have been trying to improve backbones with more layers and/or large kernels to extract more information from this dataset. This requires more computational load.

**Figure 2:**
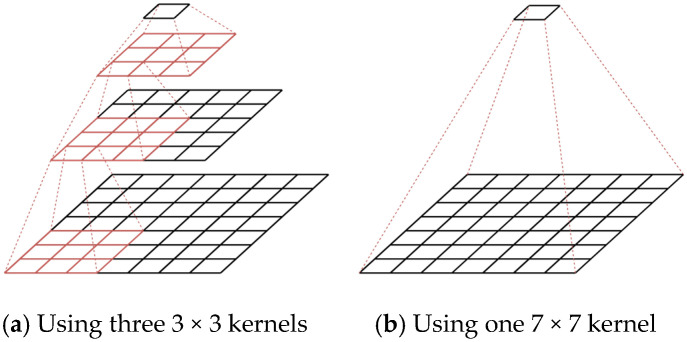
Reduction in the number of parameters by using different sized kernels.

**Figure 3:**
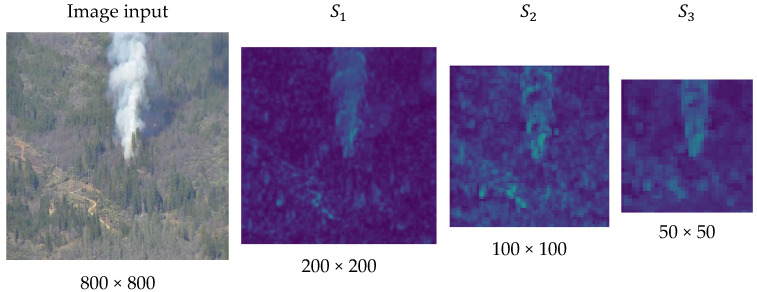
The smoke features tend to be vertically distributed through the layers.

This paper presents a new forest fire detection model to optimize smoke detection in terms of accuracy and computational load. The design of our model is fundamentally based on two principles. First, comparing the two convolution processes shown in Figure 2a,b, using a larger size kernel allows for the faster generation of feature maps but generates more parameters. Therefore, it may be advantageous to use multiple small-sized kernels to extract one feature element from the same receptive field. Second, to more effectively extract features of smoke plumes spreading along the vertical dimension, as shown in Figure 3, it may be desirable to decompose the conventional convolution with square kernels into the depth-wise convolution of coordinate kernels. This decomposition also contributes to reducing the number of parameters.

Additionally, our model extracts the features of objects through different views using kernels of different sizes to better detect smoke plumes of different scales. Our model also uses an attention mechanism that focuses on the features of specific objects (smoke) in the image while suppressing irrelevant features.

## 3. Proposed Model

### 3.1. Backbone

**Figure 4:**
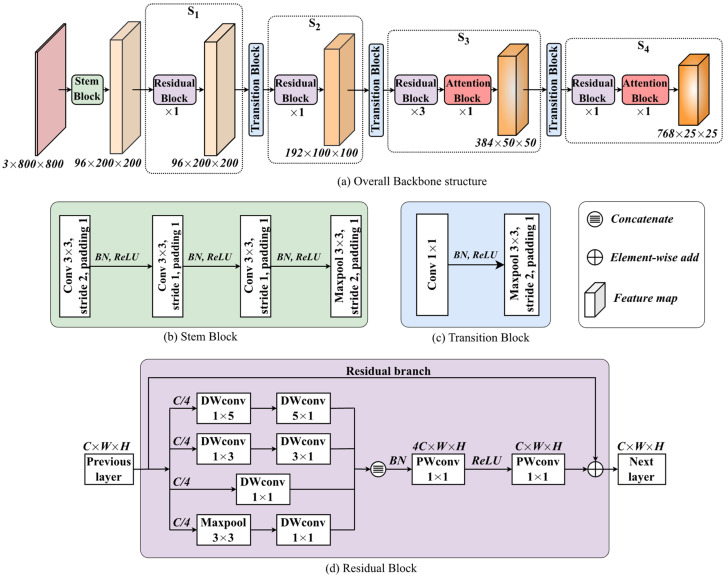
The proposed Backbone structure for forest fire detection.

The proposed backbone is structured as shown in Figure 4a. The proposed model comprehensively extracts the features of the input data by traversing a 4-stage hierarchy, where each stage consists of one or more residual blocks, with one attention block added to the residual block output of stages 3 and 4. The two design principles of the proposed backbone are to effectively extract forest smoke features to increase smoke detection accuracy and to reduce computational load. The following explains how these design principles are reflected in the structure of the proposed backbone.

#### 3.1.1. Stem Block

The stem block illustrated in Figure 4b is utilized to quickly reduce the spatial dimension of the input image without losing feature information. The stem block uses three 3 × 3 kernels with stride sizes of 2, 1, and 1 to reduce the number of parameters, while the existing ones still use a large kernel size. Even using three small kernels, the same level of information can be extracted. Like other models, Batch Normalization (BN) and the Rectified Linear Unit (ReLU) are additionally applied to the output of each convolutional layer to increase the learning speed. Note that our stem block does not use the Sigmoid Linear Unit (SiLU) and the Gaussian Error Linear Unit (GELU) functions, which consume more computational resources compared to simpler alternatives such as ReLU. At the end of the stem block, one 3×3 max pooling is applied to reduce the size of the feature map. In practice, the input image goes through four convolutional layers that use “stride 2” twice thus reducing each dimension of the feature map by a factor of four.

#### 3.1.2. Transition Block

The transition block illustrated in Figure 4c is used to shrink the size of feature map between two adjacent stages. The Conv 1 × 1 is utilized to double the number of channels, followed by 3 × 3 max pooling to reduce the spatial dimension by half. This shrinks the size of the feature map without a loss of information, while saving the number of required parameters.

#### 3.1.3. Residual Block

The residual block illustrated in Figure 4d is designed to better extract the smoke features from forest smoke. The feature map from the previous layer is split into four small feature maps along the channel dimension and each feature map is processed along different convolution layers.

The top two branches use two sequential 1×n and n×1 depth-wise convolutions (DWconv’s) instead of n×n kernels to reduce the number of parameters (*n* is given as 3 or 5 in the figure), while the third branch uses a 1 × 1 depth-wise convolution. This factorization reduces the number of parameters from n^2^ to 2n while maintaining the same receptive field. The n×1 convolutions also helps the model better capture vertically distributed features, such as smoke. The third branch can enhance feature extraction from small smoke plumes by using DWconv 1 × 1 with a small-sized kernel. The last branch sequentially applies one max pooling 3 × 3 and one DWconv 1 × 1. By taking the maximum value within each pooling region, max pooling retains the most important features while discarding less important or noisy features. One DWconv 1 × 1 is applied on the output of the max pooling layer that can help to perform channel mixing, which can improve the accuracy of the model.

The outputs of four branches are concatenated along the channel dimension to produce a fine-grained feature map, which is fed serially into two point-wise convolutions (PWconv 1 × 1 s) to mix information along the channel dimension. The ReLU activation function between them is used to reinforce functional nonlinearity in a large space via a scaling factor of four. The original feature map delivered via the residual branch is added to the resulting feature map to avoid the vanishing gradient problem [32].

#### 3.1.4. Attention Block

**Figure 5:**
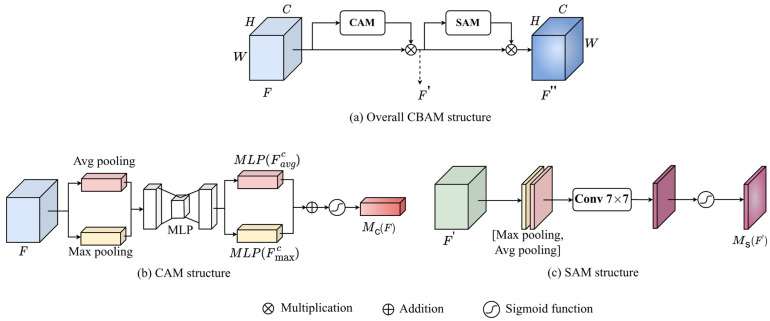
CBAM architecture.

One attention block is only added to the output of the last residual block at stages 3 and 4 as shown in Figure 4a, considering computational efficiency since the feature maps in stages 1 and 2 are large in size. The attention mechanism helps the model to focus on important features of the image while suppressing irrelevant ones. Our backbone employs the Convolution Block Attention Module (CBAM) [33] that consists of the following two components: Channel Attention Module (CAM) as shown in Figure 5b, and Spatial Attention Module (SAM) as shown in Figure 5c. In feature maps, CAM allows the model to focus on the most relevant channels, while SAM allows it to capture spatial dependencies.

Let *F* and $R^{C\times H\times W}$ represent the input feature map and a set of possible feature maps of the target object, respectively such that $F\in R^{C\times H\times W}$. Input feature map F is processed by CAM to produce channel attention weight $M_{c}(F)$ as detailed in Figure 5b. Then, the refined feature map $F^{\prime }$ is obtained by performing the element-wise matrix multiplication between $M_{c}(F)$ and F to redistribute the information in the input feature map F along the channel dimension as follows:

(1) $$F^{\prime }=M_{c}\left(F\right)⨂F.$$

Referring to Figure 5b, CAM uses average-pooling and max-pooling along the spatial dimension to aggregate the spatial information, which generate the average-pooled features $F_{avg}^{c}$ and the max-pooled features $F_{max}^{c}$, respectively. These two features are then passed to the Multilayer Perceptron (MLP) to generate two channel attention maps, $MLP(F_{avg}^{c})$ and $MLP(F_{max}^{c})$, which are merged using element-wise addition. Finally, the sigmoid function, denoted by σ, is applied to produce the channel attention weight $M_{c}\left(F\right)$ as follows:

(2) $$M_{c}\left(F\right)=\sigma \left(MLP(F_{avg}^{c})\;\;⨁\;MLP(F_{max}^{c})\right).$$

Referring to Figure 5c, the refined feature map $F^{\prime }$ is then fed into the SAM module to generate spatial attention weight $M_{s}(F\prime )$. Then, $M_{s}\left(F\prime \right)$ is multiplied with feature map $F^{\prime }$ to refine the feature map $F^{\prime }$ in a spatial dimension, thereby producing the feature map $F^{\prime \prime }$ as follows:

(3) $$F^{\prime \prime }=M_{s}\left(F^{\prime }\right)\;⨂\;F^{\prime }.$$

SAM also uses both max-pooling and average-pooling, but along the channel dimension, generates two features $F_{avg}^{s}$ and $F_{max}^{s}$ that represent the aggregated channel information. Then, they are concatenated and mixed using 7 × 7 convolution, $F^{7\times 7}$, to produce a spatial attention map. Finally, the sigmoid function σ is applied to produce the spatial attention weight $M_{s}\left(F\prime \right)$ as follows:

(4) $$M_{s}\left(F\prime \right)=\sigma \left(F^{7\times 7}(F_{avg}^{c};\;F_{max}^{c})\right).$$

Note that our model takes into account both channel attention and spatial attention since feature maps have spatial and channel dimensions.

### 3.2. Neck and Head

**Figure 6:**
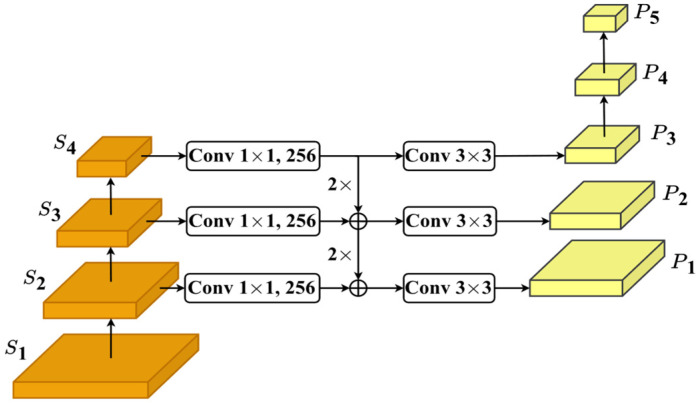
The Neck architecture.

The Neck module in Figure 6, consisting of five levels, $P_{1},\ldots ,P_{5}$, uses the slightly modified version of the Feature Pyramid Network model [30] to better detect objects of different scales as well as balance the information via multiple stages. The modifications are as follows. Initially, Conv 1 × 1 is applied to the feature maps from $S_{2}$ to $S_{4}$ in Backbone, producing a new feature map with 256 channels. The feature maps in $P_{1}$, $P_{2}$, and $P_{3}$ are produced by applying Conv 3 × 3 to the new feature map or the addition of the new feature maps, where 2× implies that up-sampling is applied twice. Note that stage $S_{1}$ is not used. The module has two more levels, $P_{4}$ and $,P_{5}$ in which feature maps are obtained by down-sampling the feature map at $P_{3}$ by 1/2 and 1/4, respectively. This can help the model to better detect larger objects.

**Figure 7:**
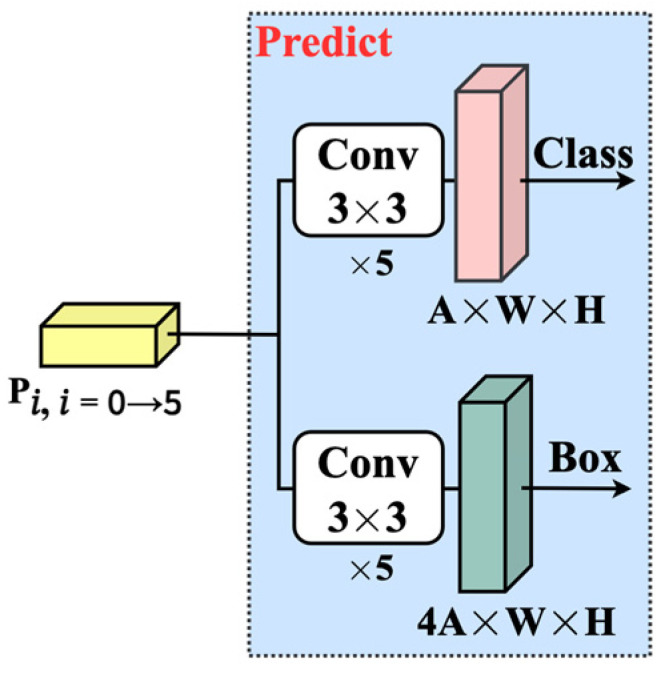
The Head architecture.

Referring to the Head module in Figure 7, object classification is represented by five Conv 3 × 3’s and one class feature map denoted by A × W × H, and bounding box regression is by five Conv 3 × 3’s and one box feature map denoted by 4A × W × H, where four indicates the four relative offset values between the anchor and the ground truth box. The anchor, inherited from RetinaNet [25], has various scales and aspect ratios to enable the model to effectively detect objects of different sizes and shapes. At each feature map location, a set of nine anchors is generated, which consists of three different scales and three aspect ratios (1:1, 2:1, 1:2). These nine anchors cover a scale range of 32 to 813 pixels with respect to the input image of the network. The anchors are applied across different levels of the Feature Pyramid Network (FPN) and allow for object detection at multiple resolutions, enabling the detection of both small and large objects.

### 3.3. Loss Function

Because forest smoke often only occupies a small region compared to the background forest area, the foreground and background classes are extremely imbalanced during training. Therefore, our model uses the focus loss (*FL*) function [25] to overcome this imbalanced training.

The focal loss function, *FL*($p_{t}$), for classification score $p_{t}$, is expressed as follows:

(5) $$FL\left(p_{t}\right)={-\left(1-p_{t}\right)}^{\gamma }\log\left(p_{t}\right)$$

where ${-\left(1-p_{t}\right)}^{\gamma }$ is the modulating factor, with tuneable focusing parameter γ = 2, and

(6) $$p_{t}=\left\{\begin{array}{ll} p & if\;y=1\\ 1-p & otherwise \end{array}\;\right.$$

where y∈{±1} specifies the ground-truth class, and p∈[0,1] is the model’s estimated probability for the class with label y=1. As suggested in the paper [20], we measure the difference between the offsets and the ground truth boxes using the bounding box regression loss function denoted by $L_{1}$. Then, the total loss, $L_{total}$, is expressed as a linear combination of $FL\left(p_{t}\right)$ and $L_{1}$ as follows:

(7) $$L_{total}=\alpha FL\left(p_{t}\right)+{\beta L}_{1},$$

where α and β are balancing terms. To determine the optimal values of the hyperparameters α, γ, and β, experiments were conducted using various α, γ, and β values recommended from the paper [25]. According to the experimental results in Table 1, the combination of α = 0.25, γ = 2, and β = 1 is known to produce the best accuracy.

## 4. Experiments

### 4.1. Dataset

There have been large-scale benchmark datasets in the object detection field, but no forest fire/smoke dataset has been found. Therefore, our primary focus is on the detection of early-stage forest fires, where the only visible sign is smoke, rather than flames. This makes our model distinct from other public fire detection benchmarks, which typically focus on detecting fire. The HPWREN dataset [34], that contains real-world imagery of early-stage forest since 2000, was considered to be somewhat suitable for our research goal of detecting smoke in the early stages of forest fires. Therefore, our initial dataset was created by extracting 2190 images from the HPWREN dataset. Our dataset was then expanded to have 4350 forest fire/smoke images by adding images from the Internet and from a forest fire surveillance system operated by a local company. However, note that since our model aims for real-time early detection of forest fires, satellite images are not currently included in the dataset due to the difficulty of real-time acquisition and insufficient image quality.

Each image in the dataset was labelled and boxed using the tool from Roboflow [35], and are then divided into a training set of 3915 images (90%) and a validation set of 435 images (10%). The dataset adequately accounted for various forest smoke scenarios by including forest smoke images varying in fire intensity, time of day, smoke shape, etc.

### 4.2. Experimental Setup

The model was implemented based on the Pytorch framework and then trained and evaluated using a computer with a GeForce RTX 3060 GPU card. The training process took 60 epochs with a batch size of 6. The learning rate was initialized as ${2.5\times 10}^{-3}$ and then decreased 10 times and 100 times after 40 epochs and 55 epochs, respectively.

Our model was compared with several existing models, including RetinaNet [25], YOLOv8 [26], YOLOv9 [27], YOLOv10 [28], Faster-RCNN [20], and SSD [29]. To ensure a fair evaluation, all models were implemented on the same dataset with consistent augmentation techniques, including flipping and rotation, to mitigate the risk of overfitting. Additionally, optimization methods, such as learning rate scheduling, optimizer selection, momentum, and weight decay, were applied across all models. Our backbone was also compared with other backbones like VGG16 [36], Convnext [37], EfficientNet [38], InceptionV1 [39], and InceptionV4 [40].

### 4.3. Evaluation Metrics

The well-known average precision (*AP*) metric from MS-COCO [41] is used to evaluate the performance of the model. AP indicates the area under a precision-recall curve, *P*(*r*), that plots the value of *precision* against *recall* for different confidence threshold values [42]. Thus, *AP* is expressed as follows:

(8) $$AP=\int_{0}^{1}P\left(r\right)dr.$$

Precision indicates the ratio of the correct predictions to all positive predictions, while Recall indicates the ratio of the correct predictions to all labeled smokes. Thus, these two metrics are expressed as follows:

(9) $$Precision=\frac{TP}{TP+FP}$$

and

(10) $$Recall=\frac{TP}{TP+FN}$$

where True Positive (TP) indicates that the model predicted the presence of smoke (Positive) and was correct (True), False Positive (FP) indicates that the model predicted the presence of smoke (Positive) but was incorrect (False), True Negative (TN) indicates that the model predicted the absence of smoke (Negative) but was correct (True), and False Negative (FN) indicates that the model predicted the absence of smoke (Negative) but was incorrect (False).

In addition to AP, some other metrics are used to evaluate the performance of the model. AP_50_ and AP_75_ indicate the AP values at 50% and 75% IoU (Intersection over Union) thresholds, respectively, and AP_S_, AP_M_, and AP_L_ are AP values for small, medium, and large objects, respectively. GFLOPs (giga floating-point operations) and #Params (the number of parameters) are used to evaluate the computational complexity of the model, and FPS (frames per second) is used to evaluate detection speed.

### 4.4. Experimental Results

According to Table 2, our model achieved the best values in AP and its sub-metrics while keeping #Params and GFLOPs low overall. RetinaNet achieved fairly competitive accuracy in AP_L_; it requires a much higher computational cost. Since RetinaNet uses a neck and head similar to our model, we can infer that the increase in computational cost comes from the backbone. Specifically, RetinaNet shows almost twice as many #Params and 6% higher GFLOPs compared to our model. The result show that our model also improves AP by approximately 9.6% while using fewer parameters and lower GFLOPs compared to Faster R-CNN, a two-stage object detection model that typically achieves high accuracy.

Meanwhile, our model requires slightly more computational load than YOLO. However, it achieves much higher accuracy, especially when detecting small objects. Looking at the APs (Average Precision Small) index, our model improves accuracy by 43.17% to 52.2% compared to the YOLO versions. Considering that the initial smoke size i s very small compared to the large monitored forest area, it seems clear that our proposed model is beneficial for early forest fire detection.

Table 3 compares the performance of the proposed backbone with other popular backbones using the same Neck, Head, and other settings. Overall, the proposed backbone achieves the best AP values while keeping fairly favorable values in #Parameters and GFLOPs. VGG16 also achieved good AP values but with significantly higher #Parameters and GFLOPs values. On the other hand, EfficientNet and InceptionV1 generated significantly fewer parameters but with significantly lower AP values of 44.0 and 41.2, respectively. It can be concluded that the proposed backbone achieves both efficiency and effectiveness for smoke detection by showing clear gains in AP and reduced computational load compared to other methods.

**Figure 8:**
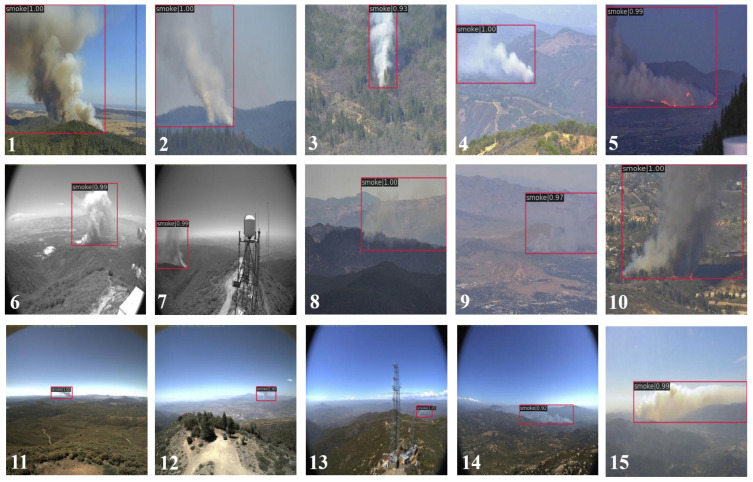
Qualitative test results for 15 forest fire images numbered 1 to 15, with the class name and confidence value given at the top of each bounding box.

Figure 8 shows the qualitative test results for 15 forest fire images numbered 1 to 15, and the class name and confidence value are given at the top of each bounding box. The proposed model was able to detect different shapes of smoke not only in the images with clear smoke such as images 1–4 but also in the monochrome or grayscale images captured by infrared cameras at night, as shown in images 6 and 7, or in the images with small or blurred smoke, like images 8, 9, and 11–14, which are difficult for humans to discern.

**Figure 9:**
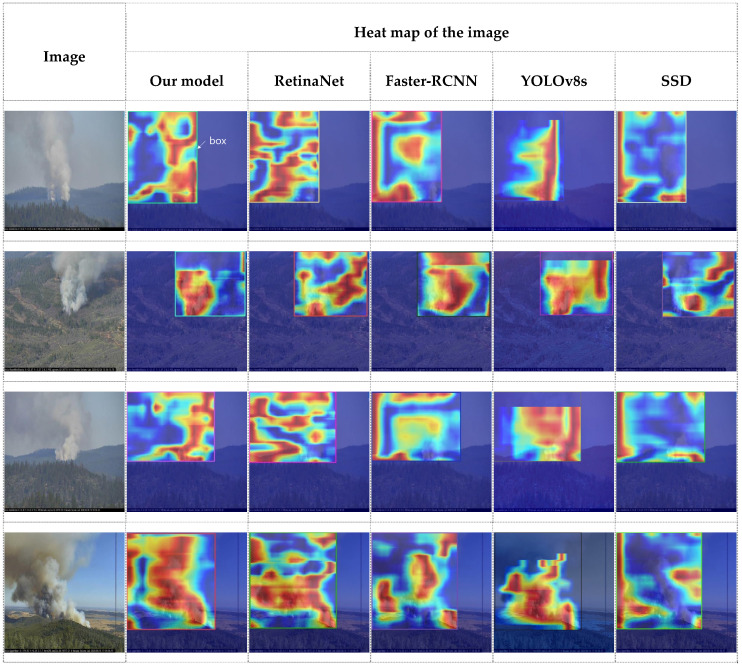
Heat maps of the images to which different models are applied.

To show how well the attention mechanism works, heat maps of the images using the Grad-CAM technique [43] with different models applied are compared in Figure 9. Note that YOLOv8s are selected as the best version of YOLO’s. The first column of the table shows four different smoke images, and each of the next five columns shows the heat map of the corresponding image when each model is applied. Looking at the heat map, hot colors such as red and yellow indicate high attention, while cool colors such as blue and green indicate low attention. It is clearly seen that the attention area of the heat map generated by our proposed model depicts the shape of the original smoke image much better compared to other heat maps. It is also seen that our model is better able to suppress the less relevant regions. For example, looking at the last smoke image in our model’s heat map, the attention region has a very similar shape to smoke.

In summary, the proposed model shows a good balance by increasing the accuracy of forest smoke detection while keeping the number of parameters to a minimum. However, due to the relatively high FLOP, more training time is required.

### 4.5. Ablation Study

Finally, we performed an ablation study to investigate the effectiveness of using techniques such as splitting (Splitting), the depth-wise convolution of coordinate kernels (DW-Coord), and an attention mechanism (CBAM) on the basic backbone (Basic) of our proposed model. According to the experimental results in Table 4, Splitting significantly reduces the number of parameters (#Params) by dividing the feature map channels into smaller segments, while DW-Coord and CBAM improve AP by helping the model focus on extracting the important features of smoke.

Consequently, the proposed model with all the techniques applied was able to increase AP by 6% while reducing #Params and GFLOPs by 34% and 14%, respectively, compared to the base model.

## 5. Conclusions

This paper presented a CNN-based forest smoke detection model featuring new backbone architecture to increase detection accuracy and reduce computational load. The backbone includes three important methods. It extracts object features through different views using kernels of varying sizes to better detect smoke plumes of different sizes. It uses the depth-wise convolution of coordinate kernels to better extract the features of smoke plumes spreading along the vertical dimension. It also employs an attention mechanism to have the model focus on important features. The model was trained using 90% of a dataset containing 4350 forest fire or smoke images and validated using 10%. According to the experimental results, our model not only improves accuracy but also reduces computational load in early forest fire detection compared to the existing models. Further research will focus on reducing FLOPs to improve learning and inference speed and optimizing the Neck and Head modules to enhance performance. The proposed model will be further examined to determine whether it can be applied to embedded systems with low computing power, such as surveillance cameras and drones.

## Figures and Tables

**Table 1:** The result of experiments using various α, γ, and β values.

γ	α	β	AP	AP_50_	AP_75_	AP_S_	AP_M_	AP_L_
0	0.75	1	48.3	79.7	46.2	22.6	44.6	80.4
0.1	0.75	1	49.4	82.8	50.2	25.3	45.4	80.5
0.2	0.75	1	50.6	84.2	47.0	25.0	47.9	84.2
0.5	0.5	1	50.2	82.9	48.0	25.8	45.3	83.8
1	0.25	1	51.6	85.6	49.8	26.7	48.8	83.8
2	0.25	1	52.9	85.7	53.3	27.8	50.2	85.8
5	0.25	1	52.3	82.8	50.4	26.6	50.3	85.0

**Table 2:** Performance comparison of our model and other models.

Model	AP	AP_50_	AP_75_	AP_S_	AP_M_	AP_L_	#Params (Millions)	GFLOPs	FPS
Our model	52.9	85.7	53.3	27.8	50.2	85.8	18.6	120.6	21.5
RetinaNet [25]	50.8	82.1	49.3	24.3	46.6	85.6	36.1	127.8	20.4
YOLOv8s [26]	49.7	77.0	51.1	14.4	45.9	77.0	11.2	28.6	102.0
YOLOv8m [26]	48.8	76.5	49.2	13.4	43.9	76.4	25.9	78.9	39.8
YOLOv9s [27]	49.1	76.1	50.6	14.0	43.1	76.6	7.1	26.4	79.5
YOLOv9m [27]	49.5	77.2	51.2	14.3	44.2	77.0	20.1	76.3	37.9
YOLOv10s [28]	48.2	75.4	48.5	15.8	40.7	75.9	7.2	21.6	88.5
YOLOv10m [28]	47.6	74.3	47.4	13.3	38.4	76.5	15.4	59.1	40.3
Faster-RCNN [20]	48.3	79.5	46.7	27.5	45.3	78.3	41.1	134.4	17.7
SSD [29]	43.0	77.8	42.4	21.6	47.1	70.8	24.4	214.2	17.4

**Table 3:** Performance comparison of proposed backbone and other backbones.

Backbone	AP	AP_50_	AP_75_	AP_S_	AP_M_	AP_L_	#Params (Millions)	GFLOPs	FPS
Our model	52.9	85.7	53.3	27.8	50.2	85.8	18.61	120.63	21.5
VGG16 [36]	49.7	83.7	48.8	25.6	45.5	82.6	142.93	331.82	12.2
Convnext [37]	48.0	81.0	46.3	19.7	45.6	78.9	19.61	90.11	22.0
EfficientNet [38]	44.0	70.9	42.0	17.2	40.7	73.1	14.58	25.75	26.1
Inceptionv1 [39]	41.2	69.4	40.4	9.6	33.8	82.1	16.13	52.25	23.8
Inceptionv4 [40]	41.0	66.4	40.3	7.5	39.2	82.0	52.92	120.43	21.0

**Table 4:** Ablation study on backbone modules with different techniques.

Basic	Splitting	DW-Coord	CBAM	AP	#Params (Million)	GFLOPs
√				49.9	28.21	140.49
√	√			50.7	20.93	125.55
√	√	√		52.6	18.52	120.62
√	√	√	√	52.9	18.61	120.63

√ indicate the inclusion of specific techniques in the backbone architecture.

## Data Availability

The data used for our experiments can be found at https://drive.google.com/drive/folders/1l9qI_EzU4A8heXvlpyGJEdxGVP3pRhcL (accessed on 8 February 2025).
